# Rapid, Sensitive and Reliable Ricin Identification in Serum Samples Using LC–MS/MS

**DOI:** 10.3390/toxins13020079

**Published:** 2021-01-22

**Authors:** Liron Feldberg, Eytan Elhanany, Orly Laskar, Ofir Schuster

**Affiliations:** 1 Department of Analytical Chemistry, Israel Institute for Biological Research, Ness Ziona 74100, Israel; 2 Department of Biochemistry and Molecular Genetics, Israel Institute for Biological Research, Ness Ziona 74100, Israel; eytanel@gmail.com; 3 Department of Infectious Diseases, Israel Institute for Biological Research, Ness Ziona 74100, Israel; orlyl@iibr.gov.il

**Keywords:** ricin, clinical samples, serum, suicide, LC–MS/MS (MRM), lactamyl-agarose, identification

## Abstract

Ricin, a protein derived from the seeds of the castor bean plant (*Ricinus communis*), is a highly lethal toxin that inhibits protein synthesis, resulting in cell death. The widespread availability of ricin, its ease of extraction and its extreme toxicity make it an ideal agent for bioterrorism and self-poisoning. Thus, a rapid, sensitive and reliable method for ricin identification in clinical samples is required for applying appropriate and timely medical intervention. However, this goal is challenging due to the low predicted toxin concentrations in bio-fluids, accompanied by significantly high matrix interferences. Here we report the applicability of a sensitive, selective, rapid, simple and antibody-independent assay for the identification of ricin in body fluids using mass spectrometry (MS). The assay involves lectin affinity capturing of ricin by easy-to-use commercial lactose–agarose (LA) beads, following by tryptic digestion and selected marker identification using targeted LC–MS/MS (Multiple Reaction Monitoring) analysis. This enables ricin identification down to 5 ng/mL in serum samples in 2.5 h. To validate the assay, twenty-four diverse naive- or ricin-spiked serum samples were evaluated, and both precision and accuracy were determined. A real-life test of the assay was successfully executed in a challenging clinical scenario, where the toxin was identified in an abdominal fluid sample taken 72 h post self-injection of castor beans extraction in an eventual suicide case. This demonstrates both the high sensitivity of this assay and the extended identification time window, compared to similar events that were previously documented. This method developed for ricin identification in clinical samples has the potential to be applied to the identification of other lectin toxins.

## 1. Introduction

Ricin, a protein derived from the seeds of the castor bean plant (*Ricinus communis)*, is the most toxic plant toxin and one of the most potent and lethal substances known. It belongs to the type 2 ribosome-inactivating proteins (RIP-II toxins), which inhibit protein synthesis, causing respiratory failure and death [1]. The protein is composed of two subunits, approximately 32 kDa each. The A subunit is an enzyme responsible for the inhibition of protein synthesis by catalyzing the depurination of a single adenosine in the 28S ribosomal subunit [2]. This reaction prevents the binding of elongation factor 2 to the ribosome and leads to protein synthesis cessation and cell death. The B subunit is a lectin, responsible for binding to galactose residues on the cell surface and for subsequent endocytosis [3]. Ricin may enter the bloodstream through a variety of exposure routes, including ingestion, injection or inhalation. The lethal dose (LD_50_) varies among these different routes; while the LD_50_ by ingestion in humans is approximately 1 mg/Kg, the LD_50_ through injection or inhalation is estimated to be three orders of magnitudes lower, as low as 1 and 3 µg/Kg, respectively [4].

The plant *Ricinus communis* is highly available since it grows wild around the world. The ricin content can be up to 1–5% (*w/w*) of the castor beans [5,6]. Ricin’s widespread availability, ease of extraction, long-term stability [7] and extreme toxicity, make it a potential agent for a bioterror attack [5,8]. According to the U.S. Centers for Disease Control and Prevention (CDC), it is classified as a Tier B bioterror agent that requires specific monitoring and necessitates improvement of diagnostic capabilities [9]. In addition to terroristic or criminal dissemination of ricin, the widespread availability of castor bean plants can lead to self-poisoning [1,6] as a result of accidental oral consumption of its seeds [6] or of intentional self-poisoning by ingestion [10,11] or injection [10,12].

Early diagnosis of ricin poisoning from clinical samples is necessary in order to take therapeutic steps to mitigate the toxicity, which is typically supportive care. An upcoming promising direction is the use of neutralizing antibodies as a post-exposure treatment for ricin intoxication [13,14], yet the therapeutic window for post-exposure intervention was found to be narrow [15], making rapid detection crucial. In a suspected bioterror event, a reliable diagnosis is highly important for correct public health decisions and surveillance [8,9]. After exposure to ricin, the toxin’s concentration in the serum or other body fluids is very low and rapidly decays. Thus, the residual ricin in poisoned rat sera was reported to be 10 ng/mL 12 h post exposure [16]. Altogether, specific, sensitive, rapid and simple analytical methods for unambiguous ricin identification from clinical samples are needed. To enable ricin identification, ricinine, a small alkaloid also found in castor bean seeds with higher levels compared to that of ricin, was suggested as a surrogate marker for ricin intoxication [10]. Indeed, the detection of ricinine in urine or serum was reported in an in vivo model for ricin intoxication [17,18,19] as measured in rat urine at least 48 h post exposure [18]. However, this approach may cause false positive results, due to the widespread use of ricinine-containing castor-oil in soaps, lubricants, dyes, pharmaceuticals, perfumes and food supplements.

Currently, ricin identification methods are mainly based on specific antibody–antigen binding, such as enzyme-linked immunosorbent assay (ELISA) or electrochemiluminescence (ECL) [20,21,22], with a limit of detection (LOD) on the ng/mL level in buffer samples. Improved sensitivity down to 5–10 pg/mL was recently achieved by an interferometry-based assay [23], or by monitoring the depurinated 28 S rRNA in a reverse-transcription-PCR assay [24,25]. Though the antibody-based approach is simple to perform, its applicability in challenging matrices such as body fluids is limited due to cross reactivity with interfering substances. Therefore, for unequivocal detection of ricin in clinical samples, direct-toxin amino acid sequence information is beneficial. Mass spectrometry (MS) is a promising method to achieve sensitive and specific ricin identification from clinical samples [26,27,28,29]. Matrix-assisted laser desorption ionization time-of-flight mass spectrometry (MALDI-TOF/MS) was reported as a method to determine intact ricin; however, its sensitivity is low and not suitable for clinical samples [30]. Improved sensitivity and specificity are achieved by high resolution MS/MS analysis of ricin tryptic peptides. This approach combines liquid chromatography (LC) retention time information with accurate masses of unique peptide markers and their fragments. This method was reported to identify ricin from several matrices, such as crude extracts, food and environmental samples with a relatively high sensitivity (ng/mL range) [26,28,31]. Usually, the LC–MS approach combines antibody-based affinity capture prior to LC–MS analysis in order to improve the assay’s sensitivity by concentrating ricin and reducing the matrix background [16]. However, the pre-capture of ricin by anti-ricin antibodies from complex matrices such as body fluids still suffers from the limitations mentioned above [32].

Recently, we developed a novel assay for ricin identification from environmental samples based on antibody-free capture using lactose–agarose (LA) beads, followed by tryptic digestion and selected marker identification by LC–MS/MS (MRM) analysis [31]. These carbohydrate beads have galactose residues that bind ricin subunit B very effectively due to its lectin character [3]. The assay was found to be sensitive (≥1 ng/mL), rapid, simple and specific for the identification of ricin in environment samples [31]. However, its applicability in clinical samples has not been demonstrated yet. Here we report the performance of this assay in clinical samples. The assay was validated in human serum samples spiked with 5 ng/mL ricin. Furthermore, it detected the toxin in a real-life clinical sample collected 72 h post injection in a suicide case.

## 2. Results

### 2.1. Adjust the Protocol to Serum

The sample preparation and analysis protocol for the assay, previously developed in our lab [31], includes the following steps: LA beads are added to a 1-mL matrix solution suspected of containing soluble toxin and rotated in an Eppendorf tube followed by several centrifugation and re-suspension washing steps. The bound toxin is resuspended in a 0.1-mL NH_4_HCO_3_ buffer, resulting in a 10-fold concentration. The bound toxin undergoes heat denaturation by a short (5 min) incubation at 95 °C resulting in a dramatic increase in its availability to the following trypsin digestion (30 min at 50 °C). The digestion process is stopped by adding formic acid. Samples analysis is performed by an LC–MS/MS (MRM) method using a triple-quadrupole coupled to ultra-performance liquid chromatography (UPLC). In environmental samples, our previously developed analysis method (taking nine minutes), successfully identified four ricin unique markers (LTTGADVR, HEIPVLPNR, LEQLAGNLR and VGLPINQR) with 6–10 MRM transitions each. The entire process takes approximately 2.5 h.

To adjust our assay for the identification of ricin from serum samples, the protocol was performed in triplicate using spiked serum previously pooled from ten naive individuals. All MRM transitions for each of the markers were evaluated to consider background noise level and interfering peaks originating in the serum matrix. The results indicated that one marker, LEQLAGNLR, presented with a high background in all transitions while the three remaining markers showed at least two transitions without interruption. Thus, the interrupted MRM transitions were excluded from the MS method to avoid false positive results. Table 1 presents a basic template of markers with their MRM transitions designed for ricin identification by the LC–MS/MS (MRM) method and a narrow panel of markers and their MRM transitions re-evaluated for the serum matrix. As can be seen in Table 1, the number of peptide markers and MRM transitions are more than what is required by the European Commission [33] for unambiguous positive identification (one unique marker with two MRM transitions). We were thus able to choose the least interrupted MRM transitions for marker identification, thereby allowing successful ricin identification in complex clinical matrices.

### 2.2. Assay Performance and Validation in Serum Samples

**Figure 1:**
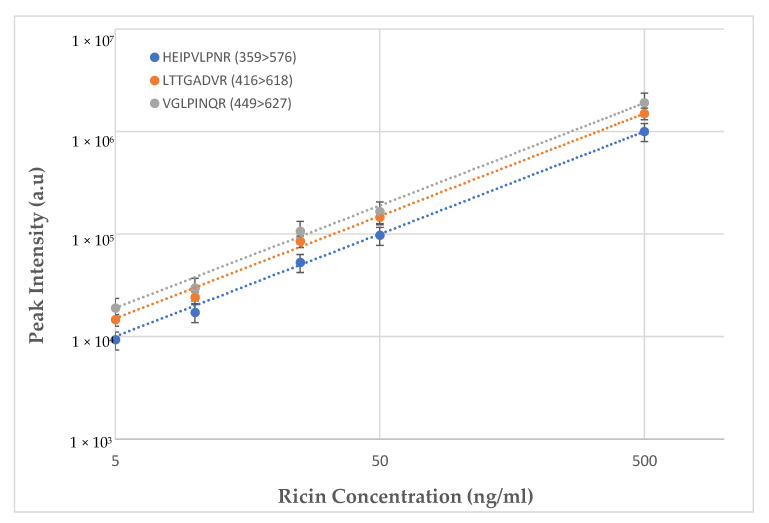
Assay linearity for the three selected tryptic peptides obtained from ricin spiked to ten pooled individuals’ serum samples, to receive final concentrations of 5–500 ng/mL.

To assess the diagnostic performance in clinical samples, ricin was spiked into a pooled serum sample derived from ten individual human samples. Test tubes were pre-blocked with a 0.5% bovine serum albumin (BSA) solution. Assays were performed in six replicates at concentrations of 0, 5, 10, 25, 50 and 500 ng/mL. The spiked samples were processed according to the described protocol, with or without dilution (in PBS) at different ratios (1:1, 1:2 and 1:4, sample:buffer, respectively). Ricin identification was achieved at concentrations as low as 5 ng/mL (with S/N = 12), and the optimal results were demonstrated at 1:1 dilution. The linearity of this assay is demonstrated in Figure 1 for the three peptide markers over a concentration range of two orders of magnitude. R^2^ values obtained for linear curves were ≥0.99. The calculated precision and accuracy (% error), for pooled human serum spiked with ricin, were lower than 30% (RSD) and 15%, respectively.

**Figure 2:**
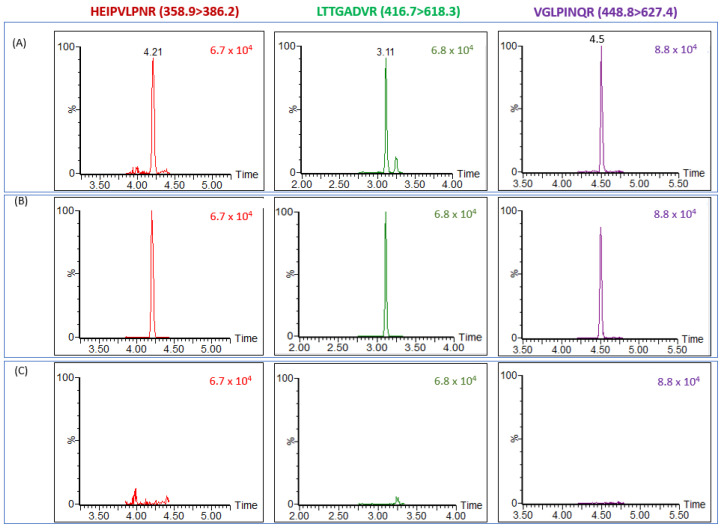
Assay validation in serum samples. LC–MS/MS (MRM) chromatograms of different samples: (**A**) Typical serum sample spiked with ricin (5 ng/mL). (**B**) Positive control: PBS spiked with ricin (5 ng/mL). (**C**) Negative control: naive serum. Each chromatogram represents the higher marker’s intensity of MRM transition.

The assay was further validated using 24 individual serum samples taken from different people. For evaluating the method’s performance, half of them were used as negative controls, while the other twelve were spiked with ricin to a final concentration of 5 ng/mL. This concentration was found to be relevant for expected ricin concentrations in serum 12 h post exposure [16]. In parallel, PBS spiked with ricin at 5 ng/mL, was used as a positive control and analyzed for evaluating the assay’s efficiency in serum-spiked samples. Ricin was unambiguously identified in all twelve spiked serum samples while no false positive result was observed in any of the negative control samples (*n* = 12). These results emphasized the reliability of our analytical method using the MRM transitions panel set for the serum matrix (Table 1). Figure 2A illustrates a representative positive individual serum sample spiked with 5 ng/mL ricin. A positive control of ricin-spiked to buffer presenting the expected ricin markers is depicted in Figure 2B while negative control (naive serum), lacking ricin markers, is shown in Figure 2C.

The identification of 5 ng/mL ricin in serum samples was based on the identification of three selected tryptic peptides with at least two MRM transitions each (Table 1). The similar peak intensities and S/N of markers obtained from spiked ricin samples, buffer and serum, indicated good efficiency of ricin capture from the serum matrix by the LA beads and emphasized the successful selection of the serum MRM transitions panel. The assay reproducibility was evaluated by calculating the precision (relative standard deviation) values for peak intensities obtained from the 12 distinct spiked serum samples. The tryptic peptide peak ratios, as well as the ratio between the MRM transitions defined for each peptide were calculated with their standard deviation. The precision values of peak ratios and intensities were calculated to be less than 20% and 30%, respectively, while the precision of MRM transitions ratios was less than 22% (Table 2). Tryptic peptides and MRM transition peak ratios were consistent with the ratios obtained from a buffer spiked with the same ricin concentrations (positive control samples).

### 2.3. Real Case Scenario

**Figure 3:**
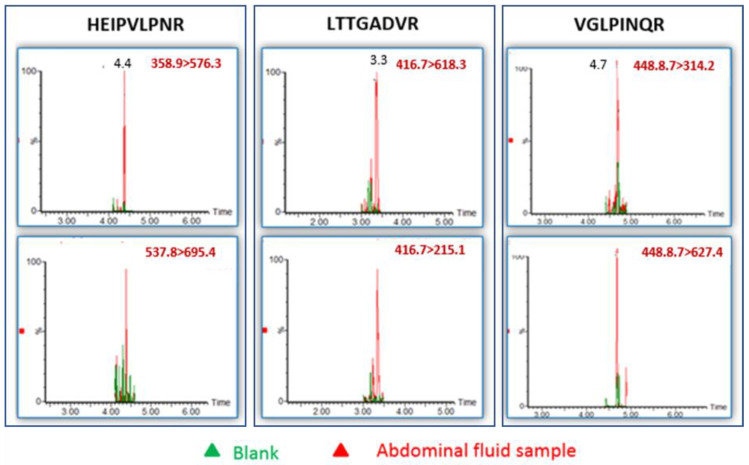
LC–MS/MS (MRM) chromatograms for the identification of ricin in abdominal fluid. The three selected peptides with their specific two MRM transitions for each peptide are presented. For each peptide (bordered by a rectangle), the upper chromatogram is for the first peptide’s transition, and the lower chromatogram is for the second.

The developed assay was applied to a challenging sample of a real-life self-poisoning event: A young person committed suicide by abdominally self-injecting a homemade castor bean extract. An abdominal fluid sample was taken 72 h post intoxication. Human serum, naive or spiked with ricin to a final concentration of 5 ng/mL, was used as negative and positive controls, respectively. (Serum was used for controls due to the unavailability of naive human abdominal fluid samples.) The samples were diluted 1:1 and analyzed according to our protocol; thus, we were able to positively identify the presence of ricin in the abdominal fluid sample (Figure 3). We further determined the ricin concentration in the sample to be approximately 3 ng/mL (by comparison with the signal intensities of 5 ng/mL ricin spiked to naive serum). Other immunoassays (i.e., ELISA) failed to detect the toxin.

## 3. Discussion

Recently we reported the development of a simple, rapid, sensitive, selective, antibody-independent MS-based assay for the identification of ricin in complex environmental samples [31]. The assay consisted of three main stages: (a) ricin capture by commercial LA beads (b) efficient tryptic digestion and (c) LC–MS/MS (MRM) analysis of specific-selected tryptic peptides. The use of LA commercial beads, an easy-to-use carbohydrate-conjugated material, is a fast and easy affinity purification tool for ricin capture that exploits the lectin-binding properties of the ricin B subunit. The capture ability of ricin from environmental samples by the LA beads was found to be very efficient (recovery > 95% for ricin up to 10 mg/mL) [31]. In environmental samples, the capture of ricin before digestion is preferable but not mandatory due to the high ricin concentrations expected in such samples. In serum samples, however, this capture is indispensable due to the low toxin concentrations expected in bio-fluids samples, accompanied by significantly high matrix interference, resulting in signal suppression. Thus, an efficient capture and concentration process is of high importance, and the use of LA beads to purify ricin from serum was found to be very efficient.

In previous work, we reported on the selection of four tryptic peptide markers (LTTGADVR, HEIPVLPNR, LEQLAGNLR and VGLPINQR) for ricin identification based on three essential criteria: selectivity, sensitivity and chemical stability. An LC–MS/MS (MRM) method, based on a triple quadrupole coupled with ultra-performance liquid chromatography (UPLC), was developed for their identification using custom-made synthetic peptides designed to correspond to the selected tryptic peptides [31]. Although, two MRM transitions are sufficient for unambiguous molecule identification according to the European criteria [33], in our method 6–10 MRM transitions from the multiple-charge molecular ions to their fragment ions were determined and optimized for each peptide in order to increase the robustness of the assay in case of unexpected complex matrix interferences. This MRM transitions panel, designed for each peptide’s marker, serves as a template to the analytical method for ricin identification. The MRM transitions, defined for each peptide, need to be re-evaluated and reselected for each matrix separately to form a reliable, not-disturbed list of MRM transitions for a particular type of matrix. Therefore, a serum-based MRM transitions panel was determined according to three main criteria: (a) no interfering peaks in negative control experiments to avoid false positives, (b) high sensitivity and (c) high specificity—the higher the *m*/*z* of the fragment, the more specific it is to the peptide. The adjusted MRM transitions panel was used for the assay and was proved to be effective according to the validation results from 24 individual serum samples. Based on our findings, we outlined three criteria for unambiguous identification of ricin in serum: (a) three tryptic peptides with at least two MRM transitions, each with S/N > 3, having a suitable MRM peak ratio as the positive control assay performed in a spiked buffer (see Table 1), (b) fixed tryptic peptide ratios similar to the positive control and (c) chromatographic retention times correlating to the positive control.

The Limit of Quantification (LOQ) for ricin spiked into human serum was as low as 5 ng/mL (S/N = 12). This concentration was reported to be within the relevant concentration for ricin analysis post ricin intoxication. For example, it was reported that 10 ng/mL of the residual ricin in poisoned rat sera could be detected 12 h post exposure [16]. Therefore, our assay appears to have the required sensitivity for the clinical diagnosis of ricin intoxication. A previous study applying a carbohydrate-enrichment approach followed by high-resolution mass spectrometry for the identification of ricin in plasma demonstrated a sensitivity of 200 ng/mL [34]. Improved sensitivity (at ng/mL levels) in rat serum was reported using an immune-capture affinity approach combined with MRM-based analysis [16]. The method presented here, combined a simple carbohydrate-capture approach using LA beads with MRM based analysis demonstrated a sensitivity comparable to the latter in human serum while being antibody independent. The method offers a simple and fast early screening of potential victims of ricin exposure by a simple serum sampling for an immediate therapeutic intervention. A real-life test of the assay was successfully executed in a challenging scenario where the toxin was identified in abdominal fluid sampled 72 h post self-injection of a castor bean extract in a suicide case. Our assay sensitivity enabled the identification of ricin from a clinical specimen that was sampled three days post exposure, thus expanding the time-window for identification [16]. To the best of our knowledge, the identification of ricin so late post exposure had not been previously reported.

## 4. Conclusions

In this study, the applicability of a simple, rapid, sensitive and selective assay for the identification of ricin in body fluids using MS was established. The assay involves ricin capture by easy-to-use commercial LA beads, based on lectin–carbohydrate affinity followed by heat denaturation, tryptic digestion of the toxin and analysis of selected markers using LC–MS/MS (MRM). To adjust the assay to a serum matrix, a reliable and uninterrupted matrix-based MRM transition panel for each marker was designed to avoid false positive results and was validated in diverse human serum samples (*n* = 24). The entire process took about 2.5 h, enabling the identification of 5 ng/mL ricin spiked into human serum. Being antibody independent, the assay can be extended to a multiplexed application for identifying the entire RIP II toxins family without skewing the result by a priori selection of target toxins by specific antibodies.

## 5. Materials and Methods

### 5.1. Reagents

All solvents and chemicals used in LC–MS/MS analysis were LC–MS grade. Water (Cat. Number 232141B1), acetonitrile (Cat. Number 120410100), and formic acid (99% purity, Cat. Number 691413) were purchased from Bio-Lab. Ammonium bicarbonate (NH_4_HCO_3_, Cat. Number A6141-500G), Lactamyl–Agarose beads (LA, Cat. Number L7634-25 mL), and octyl-β-D-glucopyranoside (OG, Cat. Number O8001-1G) were acquired from Sigma-Aldrich. Phosphate-buffered saline (PBS, pH 7.4, Cat. Number 02-023-1A), bovine serum albumin (BSA, Cat. Number 03-010-1B) and grade-modified trypsin (Cat. Number V5111) were purchased from Biological Industries. Peptides were synthesized by Merck (Sigma-Aldrich, Rehovot, Israel) with >95% purity. Normal Human Pooled Serum was purchased from MP industries (CELLect, Cat. Number 2931149).

### 5.2. Ricin Production and Sample Preparation

Crude ricin was prepared from seeds of endemic *R. communis* as described [35]. Briefly, seeds were homogenized in a Waring blender in a 5% acetic acid-phosphate buffer (CH_3_COOH-Na_2_HPO_4_, pH 7.4). The homogenate was centrifuged, and the clarified toxin-containing supernatant was subjected to ammonium sulfate precipitation (60% saturation). The precipitate was dissolved in PBS and dialyzed extensively against the same buffer. The toxin preparation was subjected to polyacrylamide gel electrophoresis and stained using Coomassie Blue, revealing two major protein bands with molecular masses of approximately 65 kDa (ricin toxin) and 120 kDa (*R. communis* agglutinin).

Blood samples were purchased from Magen David Adom (MDA), Israeli National Blood Services, as leftover whole-blood samples from unused blood donations. To separate serum from the whole blood, 5 mL whole-blood samples were centrifuged in Serum Separation Tube (BD Vacutainer^®^ SST™, ref. 456005) at 1700 g for 20 min at a temperature of 20 °C. After centrifugation, serum was collected from above the gel, and transferred to a new 15 mL tube. Adequate concentrations of ricin were spiked into the serum samples in order to receive desired concentrations, followed by dilution of the sample with PBS in a ratio of 1:1.

### 5.3. Carbohydrate Binding Using LA Beads and Tryptic Digest of the Captured Ricin

Doses of 40 µL volume of LA bead suspensions were transferred to Eppendorf tubes, followed by a short spin down (14,000 rpm, 1 min) to remove the beads’ preservation liquid. A volume of 1 mL from each clinical sample was added to the prepared LA bead tubes and vortexed vigorously, followed by a 5 min rotation to allow toxin binding to carbohydrate beads through its B subunit. The toxin, captured by LA beads, was washed twice with 50 mM ammonium bicarbonate pH 8.0 (NH_4_HCO_3_) buffer, by two centrifugation (14,000 rpm, 2 min) and resuspension cycles. Beads were then resuspended in 100 µL NH_4_HCO_3_ in the presence of 0.2% OG, and heated (95 °C, 5 min) for toxin denaturation. After a 2 min cooling, 2 µL of sequencing-grade modified trypsin (0.5 µg/µL), were added (final concentration 1 µg/100 µL) to the sample tubes, followed by 30 min incubation at 50 °C while continuously rotating. Tryptic digestion was stopped by adding 10 µL of 10% formic acid (final concentration 1%), followed by 2 min centrifugation (14,000 rpm). The resulting supernatants were transferred to LC–MS analysis vials.

### 5.4. LC/QqQ/MS-MS (MRM) Analysis

The LC–MS system consisted of Acquity UPLC-I class (SM-FTN) coupled with a Xevo TQ-S triple quadrupole mass spectrometer (Waters Corporation, Milford, MA, USA), operated with a positive ESI source in MRM mode. LC separation was performed on CSH peptides-C18 column (150 × 2.1 mm, 1.7 μm) kept at 40 °C. Mobile phases were 1% formic acid in H_2_O (A) and 1% formic acid in ACN: H_2_O (8:2 *v*/*v*, B). The gradient profile was 100% A held for 0.3 min, linearly decreased to 75% A over 4.7 min, and then decreased to 0% A over 0.1 min, held for 1.9 min, then increased to 100% A over 0.1 min and held for another 1.9 min for a total run time of 9 min. The flow rate was 0.4 mL/min, and the injection volume was 10 μL. The capillary voltage was adjusted to 0.6 kV, and the source temperature was set at 150 °C. The analytical method development process was performed using synthetic peptides as described in our previous study [31]. It included MS method development in which six to ten MRM transitions were determined and optimized for each peptide using IntelliStart software (part of MassLynx). The instrument was programed to acquire data in MRM mode. To apply the MS method to clinic samples, this basic MRM panel was re-evaluated using naive serum samples (10 pooled serum samples obtained from different donors) to form a reduced reliable, not-disturbed, list of MRM transitions (Table 1).

## Figures and Tables

**Table 1:** Basic marker template for ricin identification. The parent ion and its fragments for LC–MS/MS (MRM) analysis. The red-marked transitions were found to be uninterrupted and were therefore selected for ricin identification in the serum matrix.

Fragment *m*/*z* (Type)	Parent Ion *m*/*z* (Multiple Charge)	Tryptic Peptide
LTTGADVR	416.7 [(M + 2H)/2] ^+2^	72.1 (Imm of Val) ^+1^
		86.1 (a_3_-H_2_O) ^+2^
		187.1 (a_2_) ^+1^
		215.1 (b_2_) ^+1^
		274.2 (y_2_) ^+1^
		398.2 (a_5_-H_2_O) ^+1^
		618.3 (y_6_) ^+1^
HEIPVLPNR	537.8 [(M + 2H)/2] ^+2^	70.1 (Imm of Pro) ^+1^
		110.1 (imm of His) ^+1^
		197.6 (x_3_-H_2_O) ^+2^
		267.1 (b_2_) ^+1^
		695.4 (y_6_) ^+1^
	358.9 [(M + 3H)/3] ^+3^	70.1 (Imm of Pro) ^+1^
		86.1 (Imm of Ile) ^+1^
		345.2 (b_6_) ^+2^
		386.2 (y_3_) ^+1^
		576.3 (b_5_) ^+1^
VGLPINQR	448.8 [(M + 2H)/2] ^+2^	86.1 (Imm of Leu) ^+1^
		157.1 (b_2_) ^+1^
		183.1 (x_1_-H_2_O) ^+1^
		225.2 (a_3_-NH_3_) ^+1^
		314.2 (y_5_) ^+2^
		530.3 (y_4_) ^+1^
		627.4 (y_5_) ^+1^

**Table 2:** Assay validation in serum samples was calculated using values (peak intensities and peak intensities ratios) obtained from 12 diverse individuals serum samples spiked with 5 ng/mL ricin (diluted 1:1 with PBS). The validation was done for three tryptic peptides, two MRM transitions for LTTGADVR (416.7 > 215.1, 416.7 > 618.3), three MRM transitions for HEIPVLPNR (358.9 > 386.2, 358.9 > 576.3 and 537.8 > 695.4) and six MRM for VGLPINQR (448.8 > 314.2, 448.8 > 627.4, 448.8 > 157.1, 448.8 > 183.1, 448.8 > 225.2, 448.8 > 530.3). The table shows the validation for the two most intense MRM transitions for each peptide.

Markers	HEIPVLPNR		LTTGADVR		VGLPINQR	
Transition	359 > 576	359 > 386	416 > 215	416 > 618	449 > 314	449 > 627
Average peak intensity	33,992 ± 28%	44,233 ± 28%	20,952 ± 30%	57,125 ± 27%	48,508 ± 27%	65,675 ± 29%
Average peptide intensity ratio	1			1.7 ± 0.3	1.5 ± 0.3	
Average MRM transitions ratio	1	1.5 ± 0.3	1	2.8 ± 0.5	1	1.4 ± 0.3
